# Inhibitory Effect of *Tamarix ramosissima* Extract on the Formation of Heterocyclic Amines in Roast Lamb Patties by Retarding the Consumption of Precursors and Preventing Free Radicals

**DOI:** 10.3390/foods11071000

**Published:** 2022-03-29

**Authors:** Xiaopu Ren, Mingyang Li, Wei Wang, Xiyue Niu, Qian Xu, Ruili Zhang

**Affiliations:** 1 Xinjiang Production & Construction Group Key Laboratory of Agricultural Products Processing in Xinjiang South, College of Food Science and Engineering, Tarim University, Alar 843300, China; alarxp@vip.163.com (X.R.); lmy564181@163.com (M.L.); 2018108046@njau.edu.cn (W.W.); hljnxy0997@163.com (X.N.); 17509976820@sina.cn (Q.X.); 2 Key Laboratory of Tarim Animal Husbandry Science and Technology, Xinjiang Production & Construction Group, Tarim University, Alar 843300, China

**Keywords:** *Tamarix ramosissima* extract (TRE), heterocyclic amines (HAs), roast lamb patties, free radicals, precursors

## Abstract

*Tamarix ramosissima* has been widely used as barbecue skewers for the good taste and unique flavor it gives to the meat, but the effects of *T. ramosissima* on heterocyclic amine (HA) formation in roast lamb are unknown. The influence of *T. ramosissima* extract (TRE) on HA formation, precursors’ consumption, and free radicals’ generation in roast lamb patties were elucidated by UPLC-MS, HPLC, and electron spin resonance (ESR) analysis, respectively. Six HAs were identified and compared with the control group; the total and polar HAs decreased by 30.51% and 56.92% with TRE addition at 0.30 g/kg. The highest inhibitory effect was found against 2-amino-1-methyl-6-phenylimidazo[4,5-*f*]pyridine (PhIP) formation (70.83%) at 0.45 g/kg. The addition of TRE retarded the consumption of HA precursors, resulting in fewer HAs formed. The typical signal intensity of free radicals in roast lamb patties significantly decreased with TRE addition versus the control group (*p* < 0.05), and the higher the levels of the TRE, the greater the decrease in signal intensity. We propose that the inhibitory effects of TRE on HA formation, especially on polar HAs, were probably achieved by retarding the consumption of precursors and preventing free radicals from being generated in roast lamb patties. These findings provide valuable information concerning TRE’s effectiveness in preventing HA formation through both the precursor consumption and free radical scavenging mechanisms.

## 1. Introduction

Roast lamb is a popular food all over the world. As one of Xinjiang’s landmark culinary dishes, the lamb kebab is well known throughout China. *Tamarix ramosissima* bark is widely used as barbecue skewers and has a long history of such use; it gives the meat a good taste and flavor unique to southern Xinjiang. However, one of the problems encountered with this delicacy is that the flow of dry air in the roasting oven causes the evaporation of surface water, which means harmful compounds can form, such as heterocyclic amines (HAs) [1]. HAs are mutagenic and carcinogenic compounds, which are present at parts-per-billion (ppb) levels in the muscle of meat cooked at high temperatures. More than 30 HAs have been isolated and identified from different cooked meats [2,3,4]. According to their chemical properties, the HAs were classified as polar (imidazoquinolines, imidazoquinoxalines, and imidazopyridines) or non-polar (pyridoindole and dipyridoimidazole) [5]. Many factors affect the formation of HAs. Alaejos [2] and Gibis [3] detailed the factors that affect the varieties and amounts of HAs in meats, including the cooking conditions, type of meat, moisture content, types and amounts of HA precursors, and content of substances with enhancing or inhibiting effects on the formation of HAs. The main precursors of polar HA formation are creatine/creatinine, sugars, and amino acids originally found in meats [6], while the main precursors leading to the formation of non-polar HAs are amino acids or protein, with formation independent of the presence of creatine or creatinine [7,8]. The HAs generated through a series of complex chemical reactions that depended on these precursors, and the types and amounts of precursors, varied in different sorts of meat, especially free amino acids and creatine, which can form fundamentally different HAs [8,9]. The addition of 5% (*w*/*w*) creatine enhanced the formation of total HAs by four-fold, and the addition of amino acids (1%, *w*/*w*) increased the overall mutagenicity by between 1.5 (Lys) and 43 times (Thr) [10]. Therefore, it is expected that creatine and free amino acids were the main reactants for HA generation while roasting meats.

Accepting that a free radical mechanism is involved in HA formation, specifically polar HAs [11,12,13], to prevent or reduce HA formation, phenolic compounds have mostly been the focus of research due to their well-known free radical-scavenging properties. Cheng et al. found that the formation of polar HAs in fried beef was strongly inhibited by grape seed and apple extracts, with proanthocyanidins, phloridzin, and chlorogenic acid playing the central roles [14]. Khan et al. stated that a total of 47 phenolic compounds were identified in *Chrysanthemum morifolium* flower extract with an IC_50_ of 48.43 μg/mL against free radicals, and that phenolic compounds could be the main inhibitors of HA by reducing the total HAs up to 52% in cooked goat meat patties [15]. In our previous study, *Tamarix ramosissima* bark extract (TRE) was abundant in phenolic compounds and exhibited promising radical-scavenging activities [16]. Yet, so far, no data on the influence of the addition of TRE on the formation of HAs in roast lamb are available. In that context, this study aimed to investigate the effects of TRE on the formation and inhibition of HAs and the consumption of precursors, and whether HAs’ formation and inhibition occur through radical mechanisms, which was evaluated by electron spin resonance (ESR) studies in roast lamb.

## 2. Materials and Methods

### 2.1. Reagents and Materials

All of the chemicals and solvents were of HPLC or analytical grade. Analytical standards of 12 Has—IQ (2-amino-3-methyl-imidazo[4,5-*f*]quinoline), MeIQ (2-amino-3,4-dimethyl-imidazo[4,5-*f*]quinoline), MeIQx (2-amino-3,8-dimethyl-imidazo [4,5-*f*]quinoxaline), 4,8-DiMeIQx (2-amino-3,4,8-trimethyl-imidazo[4,5-*f*]quinoxaline), 7,8-DiMeIQx (2-amino-3,7,8-trimethyl-imidazo[4,5-*f*]quinoxaline), PhIP (2-amino-1- methyl-6-phenylimidazo[4,5-*f*]pyridine), Harman (1-methyl-9H-pyrido[3,4-*b*]indole), Norharman (9H-pyrido[3,4-*b*]indole), Trp-P-2 (3-amino-1-methyl-5H-pyrido[4,3-*b*] indole), Trp-P-1 (3-amino-1,4-dimethyl-5H-pyrido[4,3-*b*]indole), AαC (2-amino-9H- pyrido[2,3-*b*]indole), and MeAαC (2-amino-3-methyl-9H-pyrido[2,3-*b*]indole)—were supplied by Toronto Research Chemicals (Downsview, ON, Canada). The standards of free amino acids, creatine, and creatinine were purchased from Sigma-Aldrich (St. Louis, MO, USA). Oasis MCX cartridges (60 mg, 3 mL) were supplied by Waters (Milford, MA, USA). The TRE was obtained followed our previous described [16] with the extraction conditions of extraction solvent of 60% ethanol, ultrasonic-assisted extraction power of 600 W for 40 min. The vacuum freeze dryer was employed to obtain the extract powders, which were stored at −20 °C until use.

### 2.2. Preparation of Roast Lamb Patties

Lamb tender loin was obtained from a local market in Alar, China. The TRE was added to the ground lamb at 0.15, 0.30, or 0.45 g/kg and balanced for 4 h at 4 °C according to our previous study [17]. Ground lamb without additives was used as a control. Each patty (20.00 g) was formed using a petrified dish (6.0 cm diameter × 1.5 cm depth) to ensure uniformity. The patties were grilled in an electric oven (D3-256A, Toshiba, Tokyo, Japan) at 200 °C for 20 min (10 min for per side). After cooking, the patties were cooled at room temperature and cut into small pieces. The pieces of roast lamb were freeze-dried and stored at −20 °C before analysis. All experiments involving were performed in triplicate.

### 2.3. Determination of Precursors

#### 2.3.1. Total Free Amino Acid Analysis

Total free amino acid analysis was performed with an 835-50 amino acid auto-analyzer (Hitachi Co., Tokyo, Japan), and the results were expressed as mg per 100 g samples.

#### 2.3.2. Creatine and Creatinine Analysis

The contents of creatine or creatinine in the meat samples were determined using a method described by Haskaraca et al. [18], and results were expressed as milligram per gram of the sample.

#### 2.3.3. Glucose Analysis

Glucose in the roast meat samples was determined with a glucose (GO) assay kit, product number GAGO20-1KT by Sigma-Aldrich, Shanghai, China.

### 2.4. Electron Spin Resonance (ESR) Analysis for Free Radical Detection

ESR measurements were performed to evaluate the mechanisms of HA formation and reduction in roast lamb. ESR spectra were obtained using an A300-10 ESR spectrophotometer (Bruker, Rheinstetten, Germany) at room temperature. First, 0.60 g of meat samples were put into a cylindrical ESR tube (ER221/TUB4 Bruker quartz tube with a diameter of 0.5 cm) for measurement. The ESR settings were slightly modified from those described previously [19]: center field 3508 Gauss, sweep width 100 Gauss, resolution 1024 points, microwave frequency 9.84 GHz, microwave power 20 mW, time constant 164 ms, conversion time 160 ms, modulation amplitude 1.0 Gauss, modulation frequency 100 kHz, and sweep time 164 s.

### 2.5. Extraction and Determination of HAs

Twelve polar and non-polar HAs were extracted by solid-phase extraction according to Zeng et al. [20] with few modifications. Briefly, roast samples (5 g) were dissolved into 30 mL of 1 mol/L NaOH and homogenized by magnetic stirring for 2 min at room temperature. The alkaline solution was mixed with 13 g of diatomaceous earth, which was followed by adding 50 mL of ethyl acetate and ultrasonically extracted twice for 30 min. The extracts were centrifuged at 12,000× *g* for 10 min at 4 °C, and the supernatant was collected. Then, 30 mL of the ethyl acetate layer of the supernatant was transferred into Waters Oasis MCX cartridges activated by 6 mL of methanol, 6 mL of distilled water, and 6 mL of 0.1 mol/L HCl. Afterwards, the cartridges were sequentially rinsed with 6 mL of 0.1 mol/L HCl and 6 mL of methanol. The retained HAs were eluted with 6 mL of methanol–ammonia mixture (19:1, *v/v*). All of the analytes were concentrated under nitrogen flushing and dissolved in 250 μL methanol and filtrated through a 0.22 μm syringe filter just before UPLC-MS/MS analysis.

The HAs in the lamb patties were identified and quantified on an Acquity UPLC BEH C18 column (1.7 μm; 2.1 mm × 100 mm I.D.; Waters) at 35 °C. The gradient elution was achieved with a binary mobile phase of 10 mmol/L ammonium acetate (pH 6.8) (A) and acetonitrile (B). The solvent composition was 0–0.1 min, 90% A; 0.1–18 min, 10–30% B; 18–20 min, 30–100% B; 20–20.1 min, 100–10% B; and the total flow rate was 0.3 μL/min. The injection volume was 2 μL. The mass spectrometric conditions were as follows: positive ion mode; capillary voltage, 3.5 kV; ion source temperature, 120 °C; desolvation temperature, 400 °C. Data acquisition and processing were performed using Masslynx 4.1 (Waters, Milford, MA, USA). The HAs were quantified with calibration curves of each kind of HA at eight calibrant levels ranging from 0.2 to 30 ng/mL. According to Zeng et al., the determinations of LOD and LOQ were according to the signal-to-noise ratio (S/N) method, and the recovery rates for each HAs in the samples were determined by the standard addition method [20].

### 2.6. Statistical Analysis

Statistical analyses were conducted using the IBM SPSS Statistics program ver. 22 (SPSS Inc., Chicago, IL, USA). An ANOVA and Duncan’s test were used to assess the differences between different treatments. Pearson’s correlation was performed to evaluate relationships among different groups. Experiments were conducted in triplicate and data are expressed as mean ± standard deviation (SD). *p* < 0.05 was selected as the level for significant differences.

## 3. Results and Discussion

### 3.1. Effects of TRE on HAs Formation in Roast Lamb

We followed the methods for the extraction and quantification of HAs used in the study by Zeng et al. [20], which have been widely used for HAs in meat [21,22,23,24]. All 12 HAs were analyzed, but only IQ, MeIQ, PhIP, MeAαC, and the co-mutagenic β-carbolines Harman and Norharman were identified in all samples under the chosen grilling conditions. The recoveries, limit of detection (LOD), and limit of quantification (LOQ) values are given in Table S1. The average recoveries of all the HAs varied between 53.83 and 107.64%, and the LOD and LOQ values were detected to be 0.013 and 0.371 ng/g, which were parallel to those found in different studies [22,23,24].

As far as we know, no other study has evaluated the effects of TRE on HA formation in lamb during cooking. Different influences were examined of TRE on the total HA amount and individual HA levels. The contents of the six detected HAs in grilled lamb patties, expressed as ng/g dry matter, are shown in Table 1 for the addition of TRE. As can be seen from the table, the total HA contents varied between 5.58 and 8.03 ng/g. Concentrations of individual HAs ranged from 0.10 ng/g (MeIQ in lamb meat with 0.45 g/kg TRE) to 3.51 ng/g (Norharman in lamb meat with 0.45 g/kg TRE). The results are comparable with those reported by Khan et al. [15], Guo et al. [25], and Sun et al. [26]. The total for polar HAs was 2.60 ng/g for the control, and the levels of inhibition of polar HAs at the TRE levels 0.15, 0.30, and 0.45 g/kg were 53.85%, 56.92%, and 51.92%, respectively. The predominant HA found in the lamb patties for the polar fractions was IQ. In general, the addition of TRE had a significant inhibiting effect on the formation of IQ (*p* < 0.05). Compared with the control group, a significant decrease in IQ was observed with the inhibition of 63.44%, 59.68%, and 45.70% at the TRE levels 0.15, 0.30, and 0.45 g/kg (*p* < 0.05). There was no significant difference between the TRE levels 0.15 and 0.30 g/kg (*p* > 0.05), and both of the two lower TRE levels (0.15 and 0.30 g/kg) were significantly lower than the higher TRE level 0.45 g/kg (*p* < 0.05). A similar result was obtained by Guo et al. [25], where IQ was measured at 1.49 ng/g in lamb patties roasted at 200 °C for 25 min. Tengilimoglumetin et al. [27] observed that 1% hawthorn extract decreased the IQ level by 55% in pan-cooked beef at 200 °C. Oz et al. found that the inhibition of beef patties with pepper fried at 225 °C was 88.37% relative to the group without pepper [28].

As the second most abundant polar HA in the present study, PhIP is well-known as the most abundant HA formed in beef under normal cooking conditions [29]. In the present study, PhIP was also found in all cooked lamb samples, in the range of 0.14–0.48 ng/g. The levels of PhIP observed are well in accordance with the findings of Sun et al. [26]. A positive correlation was found between the level of TRE and the decrease in PhIP formation, whereby the more TRE added, the less PhIP generated. Compared with the control group, the reduction rates of PhIP were 20.83%, 50.00%, and 70.83% (*p* < 0.05) with the TRE concentration increased from 0.15 to 0.45 g/kg (Table 1). Significant differences were found between all of the groups (*p* < 0.05), indicating that TRE had an excellent reducing effect on PhIP formation. Khan et al. found a 56% reducing effect of 0.2% *Chrysanthemum morifolium* flower extract on PhIP in roasting goat patties [15]. Sabally et al. found that by adding dried apple peel extracts at 0.10%, 0.15%, and 0.30% to beef patties, PhIP was associated with inhibition by 43.13%, 59.85%, and 59.85%, respectively [30]. Furthermore, Oz et al. found the inhibition of PhIP by up to 100% with the addition of pepper to beef patties [28]. However, in another study, star anise, fennel, cumin, chili pepper, and black pepper showed no inhibitory effect on PhIP formation [22].

Similar inhibition was also observed in MeIQ, which was found to range from 0.10 to 0.26 ng/g in lamb samples. The concentrations of MeIQ decreased by 46.15%, 50.00%, and 61.54% (*p* < 0.05), respectively, after the addition of TRE at three levels compared to the control samples. It should be noted that when the addition of TRE was 0.45 g/kg, the concentrations of MeIQ were near the LOQ of the method, which was significantly lower than the other three groups (*p* < 0.05). Tengilimoglumetin et al. found that at 250 °C, hawthorn extract at the 0.5 and 1% levels decreased the formation of MeIQ by 34.33% and 44.78%, respectively [27]. When fried at 175 °C, the MeIQ in beef patties with pepper decreased by 88.06% [28].

Conversely, with the addition of the TRE, the co-mutagenic β-carbolines Harman and Norharman, which belong to the non-polar HAs, exhibited different behaviors to other HAs tested in the present study. Both were detected in all samples, and the maximum contents were 2.42 and 3.51 ng/g in the TRE group at 0.45 g/kg, respectively, indicating that the addition of TRE had a promoting effect on β-carbolines, especially on Harman. At the highest amount of TRE used in grilled lamb (0.45 g/kg), the concentrations of Harman and Norharman increased by 178.16% and 9.01% compared to the control group (*p* < 0.05), respectively. Similar results were also observed in previous research. Ahn et al. found that there were promotive or reductive effects on β-carbolines’ formation with the addition of grape seed extract, pine bark extract, and Oleoresin rosemary [31]. Several studies have also shown similar effects of different extracts on Harman and Norharman levels, including hawthorn extract [27], hibiscus extract [32], pomegranate seed extract [33], and various spices [22]. Khan et al. noted that *Chrysanthemum morifolium* flower extract inhibited the formation of Harman and Norharman by 32% and 39%, respectively, while no significant difference was found compared with the control samples [15].

As another non-polar HA, the α-carboline MeAαC, with a range of 0.69–1.34 ng/g, was different from Harman and Norharman. As the TRE levels increased, the production of MeAαC decreased by 8.21%, 43.28%, and 48.51% (*p* < 0.05), respectively. In research performed by Guo et al., 0.92 ng/g MeAαC was detected in lamb patties roasted at 200 °C for 25 min [25]. It was reported that the α-carbolines AαC and MeAαC are less well-covered in the literature compared to other HAs [27]. Similar to our results, Lee et al. found that roasted beef steaks containing extra virgin olive oil, when cooked at 200 °C, decreased the production of MeAαC from 21.44% to 55.17% with increasing levels of oils [34], while the addition of hawthorn extract to beef samples promoted the production of MeAαC [27].

Generally, TRE exhibited more effective polar HA suppression than non-polar HA in the present study, which is well in accordance with Ahn et al. [31] and Gibis et al. [35], who applied grape seed extract, pine bark extract, and Oleoresin rosemary to beef patties. Several overviews of the HA contents of red meats have been given, in which the researchers found that IQ and MeIQ range between 0 and 2 ng/g, PhIP ranges between 0 and 35 ng/g, Harman ranges between 0 and 170 ng/g, and Norharman ranges between 0 and 800 ng/g [7,36,37]. The results of the present study were all within the above-referenced HA ranges. The inhibition of HA formation in grilled lamb by TRE may be mainly due to the polyphenolics in the extract. Previously, we reported that isorhamnetin, hispidulin, cirsimaritin, and quercetin were the four main polyphenolic compounds in TRE, and other polyphenolics such as naringenin, kaempferol, apigenin, quercetin 3-*O*-glucuronide, and hesperetin were also present in the extract [16]. Zhang et al. reported that isorhamnetin, apigenin, and hesperetin all inhibited PhIP formation in a chemical model system and had a strong dose-dependent relationship, with IC_50_ values of 1.675, 0.382, and 4.251 mg/mL, respectively [38]. Cheng et al. reported that naringenin and quercetin could significantly reduce the level of PhIP relative to the control in a model and beef patties, and naringenin significantly inhibited the formation of three polar HAs (PhIP, 4,8-DiMeIQx, and MeIQx) by 70% relative to the control [39]. Zhu et al. observed that apigenin, kaempferol, quercetin, and naringenin all significantly decreased the production of Harman, Norharman, and PhIP in beef patties, and compared with the control, the total HAs were reduced by 30.96%, 46.63%, 53.74%, and 55.95%, respectively [40]. Hispidulin and quercetin 3-*O*-glucuronide from *Chrysanthemum morifolium* flower extract were considered as two of the main inhibitors of HA formation [15]. All these polyphenolics contributed to the inhibition of HAs of TRE. Polyphenolic compounds have been proven to efficiently reduce the formation of HAs in different meat products by a large number of researchers [12,15,27,31,33,35].

### 3.2. Effects of TRE on the Precursors Contents in Roast Lamb

The concentrations of different precursors—namely, free amino acids, glucose, creatine, and creatinine—are shown before and after roasting with different TRE levels in Table 2. The contents of all the free amino acids, creatine, and glucose significantly decreased after roasting (*p* < 0.05), while the creatinine concentration increased under the roasting conditions. The reduction rate for all free amino acids between the raw lamb and the roasting control group ranged from 31.96% (Gly) to 65.25% (Phe). Similarly, Gibis et al. reported that all free amino acids decreased by approximately 50% after frying [32]. It has also been revealed through several studies that the free amino acids decrease significantly in cooked meat compared to raw meat [20,24,41]. The reduction in some free amino acids is considered to be caused by the degradation of amino acids themselves or reacted with glucose, which contributes to the formation of HAs during processing [18,41].

**Figure 1:**
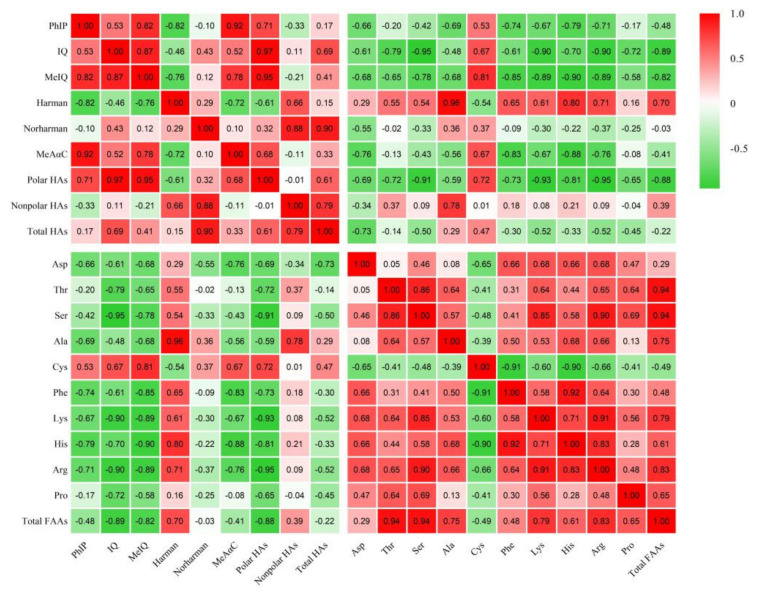
Correlation of HAs and FAAs in roast lamb patties.

The addition of different TRE levels retarded the consumption of most free amino acids, especially for Asp, Thr, Ser, Ala, Phe, Lys, His, and Arg. Compared to the control group, the total amino acids increased by 14.08%, 7.49%, and 11.21% with the addition of TRE at 0.15, 0.30, and 0.45 g/kg, respectively, and also the contents of Asp, Thr, Ser, Phe, Lys, His, and Arg significantly increased at 0.30 and 0.45 g/kg TRE (*p* < 0.05, Table 2). The increase in these free amino acids may be correlated with fewer HAs formed, except for Norharman and Harman. The correlation coefficients between the free amino acids mentioned above and different HAs were −0.73 to −0.76, −0.72 to −0.79, −0.78 to −0.95, −0.73 to −0.85, −0.89 to −0.93, −0.79 to −0.90, and −0.71 to −0.95, respectively (*p* < 0.05, Figure 1 and Table S2). Liao et al. also indicated that the correlation coefficients between Thr, Ser, Phe, and HAs were 0.91, 0.91, and 0.92, respectively [41].

Many free amino acids have been reported to be the precursors for the formation of different HAs. PhIP was produced from Phe, which was converted to phenylacetaldehyde via Strecker degradation and then reacted with creatinine, to finally form PhIP [3,42]. Some other amino acids—for example, Leu, Ile, and Tyr—have also been reported to be the precursors for the formation of PhIP [18]. Phe is further considered as a precursor of AαC and MeAαC when heated with glucose and creatinine at 100 °C for 2 h [43]. Several HAs, such as IQx, MelQx, 4,8-DiMeIQx, and 7,8-DiMeIQx, are formed from most amino acids, including Ala, Thr, Ser, Lys, Phe, and Met [44]. When amino acids such as Gly, Ala, Lys, Met, Phe, and Thr are mixed with glucose and creatinine, the HAs MeIQ, IQ, IQx, MeIQx, 4,8-DiMeIQx, 7,8-DiMeIQx, and PhIP are all formed in a model system [45].

**Figure 2:**
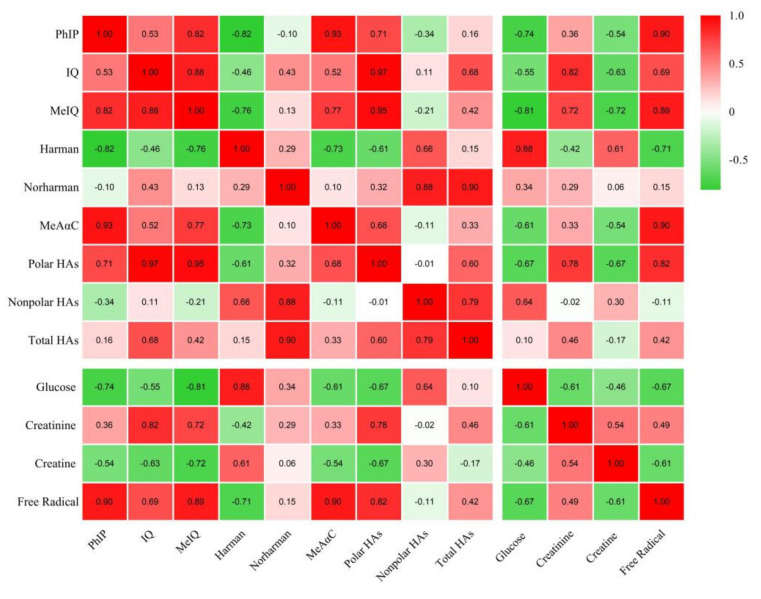
Correlation of HAs and some precursors and free radical.

The glucose concentration of raw lamb samples (1.24 mg/g) was similar to that of Gibis and Weiss, who determined the glucose level in raw lamb to be 1.82 mg/g [46]. The glucose concentrations of the control group and the groups with different levels of TRE were significantly decreased after roasting (*p* < 0.05). Similar to the results in the present study, decreased glucose levels after cooking have been reported by several researchers [18,31,32,47]. The reduction in glucose in lamb samples before and after roasting is probably due to the contribution of glucose to HAs, Maillard reactions with amino acids, and the degradation of glucose during heating [18,31]. Simultaneously, there was a significant increase in the glucose concentrations of TRE-treated samples compared with the control group (*p* < 0.05), which may be due to fewer HAs formed in the TRE group. Tengilimoglumetin et al. also observed that when artichoke extract was added to chicken breast meat samples at the 0.5 and 1.0% levels, the glucose concentrations increased significantly compared to the control group, whereas in the beef samples, similar contents were found between the artichoke extract treatment group and the control group [27]. The correlation coefficients between the glucose concentration and PhIP, MeIQ, and the total polar HAs were −0.74, −0.81 (*p* < 0.01), and −0.67 (*p* < 0.05), respectively (Figure 2 and Table S3). Harman was also correlated to the content of glucose; the correlation coefficient was determined between glucose and Harman to be r^2^ = 0.88 (*p* < 0.01). However, Norharman was not significantly associated with the glucose level (r^2^ = 0.34, *p* > 0.05). The results of the present study are in accordance with those of Gibis and Weiss, who found the same correlations between glucose concentrations and the co-mutagens Harman and Norharman [46]. Meanwhile, Gibis et al. reported correlation coefficients between glucose and different HAs of −0.76 to −0.86 [32], and Liao et al. also determined a correlation coefficient between glucose and HAs of 0.93 [41].

Creatine or creatinine in raw meat plays a key role in the mutagenic activity of HAs in cooked meats [9,18]. As can be seen in Table 2, after roasting, the content of creatine in lamb samples shows a decrease while the creatinine level shows an increasing trend. The creatine contents decreased by 31.77% to 36.89% for different groups in the present study. Similarly, a loss of range from 49.16% to 29.17% of the creatine content was reported after the cooking process [32,35,47]. A similar reduction in the creatine level of cooked meat was also reported by Haskaraca et al. [18] and Gibis et al. [47]. Jackson and Hargraves reported that in an aqueous model system, creatine levels decreased with processing time at all processing temperatures (125–250 °C), whereas creatinine levels firstly increased to a maximum value and then decreased with time; the maximum concentration of creatinine was at about 40 min of processing [48]. The reduction in creatine levels after the roasting of lamb samples compared with the raw group may be mainly due to the conversion of creatine to creatinine and the formation of HAs [18,49]. Jackson et al. observed that the conversion of creatine to creatinine was an essential step in the formation of HAs [48]. Compared to creatine, creatinine was more water-soluble and more easily transported to the patty surface with mass transport during roasting, before reacting with pyridine and pyrazine in an aldol reaction to generate HAs [3,49]. The roasting control group had lower contents of creatine and creatinine compared with groups with higher TRE addition (0.30 and 0.45 g/kg, *p* < 0.05), whereas no significant difference was found among the groups with different TRE levels (*p* > 0.05). The present results are in accordance with those of Haskaraca et al., who stated that the addition of green tea extract had different effects on the creatine and creatinine in chicken compared with the control [18].

### 3.3. ESR Analysis of Free Radicals in Roast Lamb Patties

**Figure 3:**
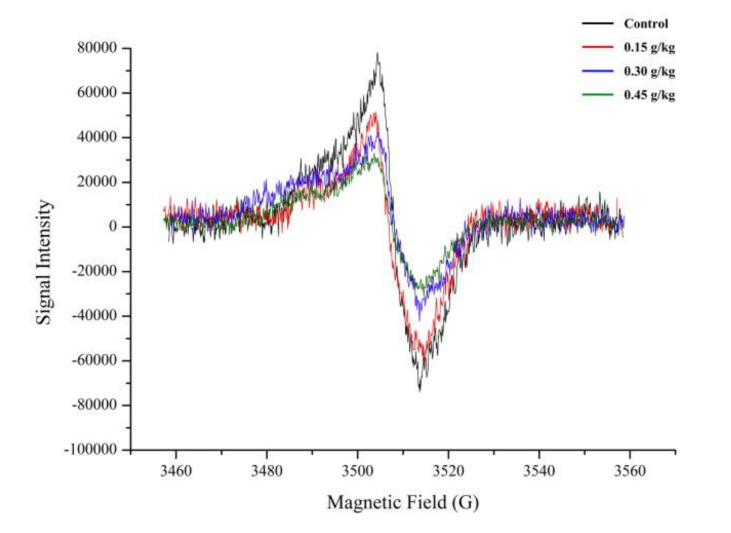
ESR spectra of roast lamb with the addition of different levels of TRE. Black curve: Control (without TRE); Red curve: 0.15 g/kg TRE; Blue curve: 0.30 g/kg TRE; Green curve: 0.45 g/kg TRE.

ESR spectroscopy is a method based on measuring the transitions of unpaired electrons in a magnetic field, and it is uniquely able to detect free radical species directly and specifically [50,51]. The magnitude of the ESR signal (peak height) indicates the distance between the maximum and minimum values of the spectral curve, and other substances have no simultaneous effect on the signal [19,52]. In the present ESR analysis, the signals of stable free radicals in roast lamb were observed, and all the roast lamb samples contained a single peak free radical signal at 3503 G of magnetic field (Figure 3, which may be the typical signal in roast lamb [53,54]. The typical signal intensity significantly decreased with the addition of TRE versus the control group (*p* < 0.05), and the higher the levels of TRE, the more the signal intensity decreased. Compared to the control group, the peak height decreased by 8.67%, 17.31%, and 18.97% with the addition of TRE at 0.15, 0.30, and 0.45 g/kg, respectively. The correlation between free radicals and all the polar HAs in the present study was strongly significant, and the coefficients were 0.90, 0.69, and 0.89 between free radicals and PhIP, IQ, and MeIQ (*p* < 0.01, Figure 2 and Table S3), respectively. Conversely, no significant difference was found between free radicals and non-polar HAs (*p* > 0.05). In other words, both the amounts of HA and ESR signal intensity decreased with the addition of TRE.

In our previous study, TRE exhibited excellent free radical scavenging ability in vitro, and the phenolic compounds in TRE, such as isorhamnetin, cirsimaritin, quercetin, and kaempferol, were all proven to be good free radical scavenging agents [16]. The remarkable inhibition properties on HAs and free radicals may be related to the abundant phenolic compounds in TRE. The results corroborate with other data previously published on HA formation and free radical reduction, especially concerning polar HAs’ formation. Kikugawa elucidated that three phenolic antioxidants, butylated hydroxyanisole (BHA), sesamol, and epigallocatechin gallate (EGCG), could reduce the formation of imidazoquinoxaline-type HAs in cooking and meat processing by preventing the generation of unstable free radicals or destroying the radicals, as demonstrated by ESR analysis [12]. Tsen et al. demonstrated that antioxidants acted as free radical scavengers in the early stages of the Maillard reaction by inhibiting the free radical intermediate form of the pyridines and/or pyrazine, and finally inhibiting polar HAs’ formation [55]. Similar conclusions have been supported by many researchers, i.e., that the natural extracts and phenolic antioxidants effectively inhibit the formation of polar HAs by scavenging the free radical mechanism [27,30,31,35,56]. Several researchers have identified the free radicals formed in the early Maillard reaction in the model system. Tetsuta et al. demonstrated that three kinds of free radicals are involved in HAs’ formation, which were found in the heated model system composed of glucose/glycine/creatinine, including the unstable free radical Maillard intermediates, the pyrazine cation radical, and certain carbon-centered radical(s). HAs’ generation was reduced by polyphenolics compounds mainly by preventing or destroying the pyrazine cation radical and the carbon-centered radical(s) [11]. Kikugawa also considered the pyrazine cation radical as a prerequisite for the formation of HAs, especially for imidazoquinoxaline-type HAs, and proposed that it is critical to prevent its generation or scavenge it in the early stages of the Maillard reaction to decrease HAs’ formation [10].

However, several researchers hold the opposite opinions that there is no positive correlation between the inhibitory activity of phenolic compounds on HA formation and free radical scavenging activities, and they consider that the mechanism of phenolic compounds’ inhibition of HAs’ formation is more complex than just free radical scavenging [39,57]. In recent years, several new inhibition mechanisms of HAs have been proposed, including trapping or scavenging intermediates named reactive carbonyl species (RCS) to form adducts, competitive inhibition of precursors, and elimination of end-products [17,40,58,59]. Due to the complexity and variability of the roast lamb system, and HAs’ formation process, the free radical-scavenging mechanism and some other mechanisms are suggested to occur simultaneously, which may have a synergistic effect preventing the formation of HAs.

## 4. Conclusions

Generally, the results of the present study have shown that the addition of TRE can effectively inhibit the formation of HAs, especially polar HAs, in lamb patties roasted at 200 °C. Most of the precursors’ consumption was retarded, and the typical signal intensity of free radicals was remarkably decreased with the addition of TRE. The possible mechanism for the inhibition of HAs of TRE aligns well with the previously proposed mechanism involving unstable free radical Maillard intermediates’ reactions, and the phenolic antioxidants in TRE acted as strong scavengers of free radical species. These findings may provide a basis for the wider use of *T. ramosissima* in barbecue skewers to improve their flavor and inhibit the formation of HAs during processing.

## Figures and Tables

**Table 1:** Inhibition effects of TRE on HAs formation in roast lamb.

HAs		Control	TRE Levels		
			0.15 g/kg	0.30 g/kg	0.45 g/kg
Polar HAs	IQ	1.86 ± 0.08 ^a^	0.68 ± 0.04 ^c^	0.75 ± 0.04 ^c^	1.01 ± 0.06 ^b^
	MeIQ	0.26 ± 0.01 ^a^	0.14 ± 0.01 ^b^	0.13 ± 0.01 ^b^	0.10 ± 0.01 ^c^
	PhIP	0.48 ± 0.04 ^a^	0.38 ± 0.04 ^b^	0.24 ± 0.03 ^c^	0.14 ± 0.02 ^d^
Nonpolar HAs	Harman	0.87 ± 0.08 ^c^	1.48 ± 0.28 ^b^	1.37 ± 0.17 ^b^	2.42 ± 0.33 ^a^
	Norharman	3.22 ± 0.18 ^ab^	2.88 ± 0.23 ^bc^	2.34 ± 0.29 ^c^	3.51 ± 0.42 ^a^
	MeAαC	1.34 ± 0.07 ^a^	1.23 ± 0.08 ^b^	0.76 ± 0.04 ^c^	0.69 ± 0.04 ^c^
Total HAs		8.03 ± 0.29 ^a^	6.79 ± 0.26 ^b^	5.58 ± 0.12 ^c^	7.87 ± 0.29 ^a^
Inhibition(%)			−15.44	−30.51	−1.99

Note: Means with different superscripts within the same row are significantly different at *p* < 0.05. Values are represented as means ± standard deviation (SD).

**Table 2:** Content of different precursors in roast lamb with different levels of TRE.

Precursors mg/100 g	Raw Lamb	Roast Control	TRE Levels		
			0.15 g/kg	0.30 g/kg	0.45 g/kg
Asp	3.26 ± 0.05 ^a^	1.58 ± 0.12 ^d^	1.73 ± 0.02 ^cd^	2.12 ± 0.11 ^b^	1.86 ± 0.01 ^c^
Thr	61.97 ± 2.22 ^a^	25.07 ± 1.14 ^e^	40.27 ± 0.46 ^b^	29.95 ± 0.76 ^cd^	33.86 ± 0.21 ^c^
Ser	16.00 ± 1.41 ^a^	8.65 ± 0.22 ^c^	10.29 ± 0.34 ^b^	9.83 ± 0.19 ^b^	9.73 ± 0.13 ^b^
Glu	26.17 ± 1.97 ^a^	14.34 ± 0.31 ^b^	14.65 ± 0.56 ^b^	14.13 ± 0.13 ^b^	13.61 ± 0.51 ^b^
Gly	21.11 ± 1.49 ^a^	14.36 ± 0.00 ^b^	13.90 ± 0.47 ^b^	14.32 ± 0.29 ^b^	14.19 ± 0.37 ^b^
Ala	78.58 ± 2.66 ^a^	47.72 ± 1.24 ^d^	52.32 ± 1.19 ^c^	48.89 ± 1.07 ^d^	55.95 ± 0.81 ^b^
Cys	1.66 ± 0.37 ^a^	0.82 ± 0.00 ^b^	0.75 ± 0.08 ^b^	0.72 ± 0.00 ^b^	0.72 ± 0.00 ^b^
Val	15.72 ± 1.07 ^a^	7.54 ± 0.22 ^b^	8.04 ± 0.91 ^b^	7.86 ± 0.10 ^b^	6.91 ± 0.19 ^b^
Met	14.07 ± 0.99 ^a^	5.70 ± 0.09 ^b^	5.70 ± 0.74 ^b^	5.84 ± 0.03 ^b^	5.16 ± 0.11 ^b^
Ile	9.85 ± 0.98 ^a^	5.87 ± 0.03 ^bc^	5.43 ± 0.16 ^c^	5.99 ± 0.22 ^bc^	6.16 ± 0.47 ^b^
Leu	26.48 ± 1.99 ^a^	12.85 ± 0.07 ^b^	13.72 ± 0.69 ^b^	13.03 ± 0.22 ^b^	11.66 ± 0.34 ^c^
Tyr	11.88 ± 0.29 ^a^	6.87 ± 0.12 ^b^	7.30 ± 0.42 ^b^	7.13 ± 0.46 ^b^	6.48 ± 0.16 ^b^
Phe	26.13 ± 0.43 ^a^	8.70 ± 0.23 ^c^	10.19 ± 1.24 ^bc^	11.39 ± 0.47 ^b^	11.87 ± 0.55 ^b^
Lys	19.89 ± 0.38 ^a^	8.53 ± 0.12 ^c^	9.04 ± 0.14 ^b^	9.08 ± 0.07 ^b^	9.03 ± 0.18 ^b^
His	7.93 ± 0.02 ^a^	4.15 ± 0.12 ^d^	4.85 ± 0.41 ^c^	5.21 ± 0.14 ^bc^	5.55 ± 0.19 ^b^
Arg	28.04 ± 0.32 ^a^	11.07 ± 0.24 ^c^	12.33 ± 0.26 ^b^	12.46 ± 0.30 ^b^	12.48 ± 0.34 ^b^
Pro	19.56 ± 1.73 ^a^	8.63 ± 0.19 ^b^	9.02 ± 0.02 ^b^	8.91 ± 0.08 ^b^	8.78 ± 0.18 ^b^
Total free amino acid	388.32 ± 7.97 ^a^	192.44 ± 2.55 ^d^	219.54 ± 3.83 ^b^	206.85 ± 0.17 ^c^	214.01 ± 1.03 ^b^
Creatine mg/g	11.52 ± 0.62 ^a^	7.86 ± 0.76 ^b^	7.45 ± 0.08 ^bc^	7.27 ± 0.14 ^c^	7.27 ± 0.26 ^c^
Creatinine mg/g	0.08 ± 0.00 ^c^	0.66 ± 0.09 ^a^	0.47 ± 0.06 ^b^	0.51 ± 0.02 ^b^	0.50 ± 0.04 ^b^
Glucose mg/g	1.24 ± 0.07 ^a^	0.55 ± 0.04 ^e^	0.72 ± 0.02 ^c^	0.64 ± 0.03 ^d^	0.78 ± 0.02 ^b^

Note: Means with different superscripts within the same row (a–e) are significantly different at *p* < 0.05. Values are represented as means ± standard deviation (SD).

## Data Availability

The data presented in this study are available on request from the corresponding author.

## Supplementary Materials

The following supporting information can be downloaded at: https://www.mdpi.com/article/10.3390/foods11071000/s1, Table S1: Analytical characteristics of HA standard solutions, Table S2: The correlation of HAs and free amino acid, Table S3: The correlation of HAs and some precursors and free radical.
