# Supplementary material for: Inhibitory Effect of Tamarix ramosissima Extract on the Formation of Heterocyclic Amines in Roast Lamb Patties by Retarding the Consumption of Precursors and Preventing Free Radicals
Source: Foods. 2022 Mar 29;11(7):1000. doi: 10.3390/foods11071000 (PMC8998052; doi:10.3390/foods11071000)
Supplement: Supplementary file 1 [file foods-11-01000-s001.zip › foods-1632029-supplementary.pdf]

**Table S1** Analytical characteristics of HA standard solutions

| HAs | Linear range (ng/mL) | Calibration curve | Coefficients ($R^2$) | LOD (ng/g) | LOQ (ng/g) | RSD (%) | Recovery (%) |
|---|---|---|---|---|---|---|---|
| PhIP | 0.4-66.6 | y=118.92x−33.97 | 0.9982 | 0.080 | 0.112 | 9.86 | 73.40 |
| IQ | 0.9-110.3 | y=23.65x−10.99 | 0.9985 | 0.044 | 0.073 | 7.31 | 71.32 |
| MeIQ | 0.2-98.5 | y=27.80x+1.95 | 0.9921 | 0.054 | 0.091 | 8.52 | 69.81 |
| 8-MeIQx | 0.3-72.2 | y=109.04x−25.04 | 0.9988 | 0.089 | 0.122 | 8.82 | 59.71 |
| 4,8-DiMeIQx | 0.3-77.3 | y=377.22x−414.61 | 0.9956 | 0.013 | 0.024 | 8.82 | 73.18 |
| 7,8-DiMeIQx | 0.3-54.1 | y=376.57x−370.55 | 0.9954 | 0.205 | 0.371 | 12.22 | 53.83 |
| AαC | 0.3-69.2 | y=114.00x−31.49 | 0.9978 | 0.019 | 0.050 | 6.73 | 78.24 |
| MeAαC | 0.3-54.5 | y=167.17x−71.67 | 0.9993 | 0.021 | 0.039 | 9.15 | 81.75 |
| Harman | 0.6-53.7 | y=188.72x−67.55 | 0.9930 | 0.052 | 0.082 | 7.24 | 96.42 |
| Norharman | 0.3-62.1 | y=142.11x−41.93 | 0.9983 | 0.109 | 0.133 | 5.32 | 107.64 |
| Trp-P-1 | 0.3-69.0 | y=163.04x−63.03 | 0.9964 | 0.080 | 0.197 | 8.75 | 60.85 |
| Trp-P-2 | 0.2-70.5 | y=93.76x−25.91 | 0.9977 | 0.108 | 0.152 | 7.53 | 54.23 |

Note: LOD means Limit of detection. LOQ means Limit of quantitation.

**Table S2** The correlation of HAs and free amino acid

| Correlation | PhIP | IQ | MeIQ | Harman | Norharman | MeA$\alpha$C | Polar HAs | Nonpolar HAs | Total HAs | Asp | Thr | Ser | Glu | Gly | Ala | Cys | Val | Met | Ile | Leu | Tyr | Phe | Lys | His | Arg | Pro | TotalFAA |
|---|---|---|---|---|---|---|---|---|---|---|---|---|---|---|---|---|---|---|---|---|---|---|---|---|---|---|---|
| PhIP | 1 | 0.531 | .824** | -.817** | -0.096 | .923** | .710** | -0.332 | 0.17 | -0.659 | -0.198 | -0.421 | 0.571 | -0.186 | -0.689 | 0.529 | 0.288 | 0.27 | -0.566 | 0.566 | 0.353 | -.736* | -0.672 | -.791* | -.707* | -0.175 | -0.479 |
| IQ | 0.531 | 1 | .869** | -0.463 | 0.433 | 0.517 | .973** | 0.114 | .686* | -0.611 | -.794* | -.951** | -0.005 | 0.387 | -0.479 | 0.673 | -0.232 | -0.002 | 0.165 | -0.201 | -0.312 | -0.614 | -.897** | -0.7 | -.903** | -.719* | -.888** |
| MeIQ | .824** | .869** | 1 | -.764** | 0.117 | .776** | .949** | -0.213 | 0.411 | -0.682 | -0.652 | -.780* | 0.393 | 0.295 | -0.678 | .813* | 0.138 | 0.309 | -0.172 | 0.237 | 0.053 | -.853** | -.887** | -.903** | -.890** | -0.58 | -.816* |
| Harman | -.817** | -0.463 | -.764** | 1 | 0.292 | -.722** | -.612* | .663* | 0.153 | 0.287 | 0.545 | 0.541 | -0.534 | -0.202 | .964** | -0.541 | -0.45 | -0.566 | 0.208 | -0.587 | -0.478 | 0.65 | 0.614 | .805* | .714* | 0.165 | 0.7 |
| Norharman | -0.096 | 0.433 | 0.117 | 0.292 | 1 | 0.099 | 0.325 | .877** | .896** | -0.546 | -0.02 | -0.329 | -0.064 | 0.165 | 0.357 | 0.372 | -0.294 | -0.232 | 0.433 | -0.295 | -0.202 | -0.093 | -0.3 | -0.217 | -0.37 | -0.245 | -0.026 |
| MeA$\alpha$C | .923** | 0.517 | .776** | -.722** | 0.099 | 1 | .678* | -0.112 | 0.325 | -.759* | -0.13 | -0.43 | 0.606 | -0.116 | -0.561 | 0.669 | 0.347 | 0.295 | -0.534 | 0.573 | 0.36 | -.827* | -0.665 | -.876** | -.764* | -0.077 | -0.412 |
| Polar HAs | .710** | .973** | .949** | -.612* | 0.325 | .678* | 1 | -0.006 | .607* | -0.69 | -.725* | -.910** | 0.163 | 0.287 | -0.593 | .721* | -0.103 | 0.089 | -0.016 | -0.006 | -0.158 | -.725* | -.935** | -.810* | -.948** | -0.654 | -.877** |
| Nonpolar HAs | -0.332 | 0.114 | -0.213 | .663* | .877** | -0.112 | -0.006 | 1 | .792** | -0.343 | 0.368 | 0.088 | -0.256 | -0.096 | .776* | 0.014 | -0.41 | -0.48 | 0.244 | -0.444 | -0.373 | 0.182 | 0.085 | 0.214 | 0.09 | -0.041 | 0.394 |
| Total HAs | 0.17 | .686* | 0.411 | 0.153 | .896** | 0.325 | .607* | .792** | 1 | -.726* | -0.142 | -0.498 | -0.116 | 0.098 | 0.289 | 0.465 | -0.414 | -0.353 | 0.198 | -0.382 | -0.418 | -0.301 | -0.515 | -0.327 | -0.52 | -0.446 | -0.215 |
| Asp | -0.659 | -0.611 | -0.682 | 0.287 | -0.546 | -.759* | -0.69 | -0.343 | -.726* | 1 | 0.048 | 0.457 | -0.313 | 0.194 | 0.081 | -0.648 | 0.11 | 0.099 | 0.31 | -0.119 | 0.017 | 0.658 | 0.676 | 0.664 | 0.68 | 0.468 | 0.285 |
| Thr | -0.198 | -.794* | -0.652 | 0.545 | -0.02 | -0.13 | -.725* | 0.368 | -0.142 | 0.048 | 1 | .863** | 0.162 | -0.623 | 0.638 | -0.411 | 0.156 | -0.168 | -0.447 | 0.256 | 0.262 | 0.311 | 0.644 | 0.442 | 0.648 | 0.645 | .944** |
| Ser | -0.421 | -.951** | -.780* | 0.541 | -0.329 | -0.43 | -.910** | 0.088 | -0.498 | 0.457 | .863** | 1 | 0.159 | -0.341 | 0.568 | -0.482 | 0.337 | 0.082 | -0.311 | 0.279 | 0.317 | 0.411 | .852** | 0.585 | .897** | 0.688 | .941** |
| Glu | 0.571 | -0.005 | 0.393 | -0.534 | -0.064 | 0.606 | 0.163 | -0.256 | -0.116 | -0.313 | 0.162 | 0.159 | 1 | 0.229 | -0.355 | 0.59 | .813* | .732* | -0.367 | .876** | 0.706 | -0.621 | -0.23 | -0.638 | -0.209 | 0.303 | 0.064 |
| Gly | -0.186 | 0.387 | 0.295 | -0.202 | 0.165 | -0.116 | 0.287 | -0.096 | 0.098 | 0.194 | -0.623 | -0.341 | 0.229 | 1 | -0.242 | 0.454 | 0.241 | 0.469 | 0.595 | -0.041 | -0.072 | -0.21 | -0.315 | -0.284 | -0.212 | -0.185 | -0.416 |
| Ala | -0.689 | -0.479 | -0.678 | .964** | 0.357 | -0.561 | -0.593 | .776* | 0.289 | 0.081 | 0.638 | 0.568 | -0.355 | -0.242 | 1 | -0.392 | -0.383 | -0.523 | 0.088 | -0.473 | -0.41 | 0.498 | 0.527 | 0.677 | 0.657 | 0.129 | .748* |
| Cys | 0.529 | 0.673 | .813* | -0.541 | 0.372 | 0.669 | .721* | 0.014 | 0.465 | -0.648 | -0.411 | -0.482 | 0.59 | 0.454 | -0.392 | 1 | 0.439 | 0.588 | -0.101 | 0.4 | 0.291 | -.911** | -0.597 | -.903** | -0.658 | -0.413 | -0.49 |
| Val | 0.288 | -0.232 | 0.138 | -0.45 | -0.294 | 0.347 | -0.103 | -0.41 | -0.414 | 0.11 | 0.156 | 0.337 | .813* | 0.241 | -0.383 | 0.439 | 1 | .928** | -0.351 | .914** | .860** | -0.494 | 0.185 | -0.462 | 0.054 | 0.489 | 0.144 |
| Met | 0.27 | -0.002 | 0.309 | -0.566 | -0.232 | 0.295 | 0.089 | -0.48 | -0.353 | 0.099 | -0.168 | 0.082 | .732* | 0.469 | -0.523 | 0.588 | .928** | 1 | -0.113 | .790* | .789* | -0.553 | 0.03 | -0.568 | -0.105 | 0.197 | -0.129 |

| | | | | | | | | | | | | | | | | | | | | | | | | | | | |
|---|---|---|---|---|---|---|---|---|---|---|---|---|---|---|---|---|---|---|---|---|---|---|---|---|---|---|---|
| Ile | -0.566 | 0.165 | -0.172 | 0.208 | 0.433 | -0.534 | -0.016 | 0.244 | 0.198 | 0.31 | -0.447 | -0.311 | -0.367 | 0.595 | 0.088 | -0.101 | -0.351 | -0.113 | 1 | -0.521 | -0.289 | 0.465 | -0.063 | 0.224 | -0.047 | -0.229 | -0.212 |
| Leu | 0.566 | -0.201 | 0.237 | -0.587 | -0.295 | 0.573 | -0.006 | -0.444 | -0.382 | -0.119 | 0.256 | 0.279 | .876** | -0.041 | -0.473 | 0.4 | .914** | .790* | -0.521 | 1 | .917** | -0.526 | 0.039 | -0.548 | -0.099 | 0.48 | 0.127 |
| Tyr | 0.353 | -0.312 | 0.053 | -0.478 | -0.202 | 0.36 | -0.158 | -0.373 | -0.418 | 0.017 | 0.262 | 0.317 | 0.706 | -0.072 | -0.41 | 0.291 | .860** | .789* | -0.289 | .917** | 1 | -0.32 | 0.234 | -0.413 | -0.003 | 0.465 | 0.191 |
| Phe | -.736* | -0.614 | -.853** | 0.65 | -0.093 | -.827* | -.725* | 0.182 | -0.301 | 0.658 | 0.311 | 0.411 | -0.621 | -0.21 | 0.498 | -.911** | -0.494 | -0.553 | 0.465 | -0.526 | -0.32 | 1 | 0.582 | .916** | 0.642 | 0.303 | 0.479 |
| Lys | -0.672 | -.897** | -.887** | 0.614 | -0.3 | -0.665 | -.935** | 0.085 | -0.515 | 0.676 | 0.644 | .852** | -0.23 | -0.315 | 0.527 | -0.597 | 0.185 | 0.03 | -0.063 | 0.039 | 0.234 | 0.582 | 1 | .714* | .906** | 0.565 | .790* |
| His | -.791* | -0.7 | -.903** | .805* | -0.217 | -.876** | -.810* | 0.214 | -0.327 | 0.664 | 0.442 | 0.585 | -0.638 | -0.284 | 0.677 | -.903** | -0.462 | -0.568 | 0.224 | -0.548 | -0.413 | .916** | .714* | 1 | .831* | 0.284 | 0.614 |
| Arg | -.707* | -.903** | -.890** | .714* | -0.37 | -.764* | -.948** | 0.09 | -0.52 | 0.68 | 0.648 | .897** | -0.209 | -0.212 | 0.657 | -0.658 | 0.054 | -0.105 | -0.047 | -0.099 | -0.003 | 0.642 | .906** | .831* | 1 | 0.484 | .827* |
| Pro | -0.175 | -.719* | -0.58 | 0.165 | -0.245 | -0.077 | -0.654 | -0.041 | -0.446 | 0.468 | 0.645 | 0.688 | 0.303 | -0.185 | 0.129 | -0.413 | 0.489 | 0.197 | -0.229 | 0.48 | 0.465 | 0.303 | 0.565 | 0.284 | 0.484 | 1 | 0.648 |
| Total FAA | -0.479 | -.888** | -.816* | 0.7 | -0.026 | -0.412 | -.877** | 0.394 | -0.215 | 0.285 | .944** | .941** | 0.064 | -0.416 | .748* | -0.49 | 0.144 | -0.129 | -0.212 | 0.127 | 0.191 | 0.479 | .790* | 0.614 | .827* | 0.648 | 1 |

**Table S3** The correlation of HAs and some precursors and free radical

| Correlation | PhIP | IQ | MeIQ | Harman | Norharman | MeAαC | Polar HAs | Nonpolar HAs | Total HAs | Glucose | Creatinine | Creatine | Free radical |
|---|---|---|---|---|---|---|---|---|---|---|---|---|---|
| PhIP | 1 | 0.531 | .824** | -.817** | -0.096 | .923** | .710** | -0.332 | 0.17 | -.740** | 0.374 | -0.532 | .900** |
| IQ | 0.531 | 1 | .869** | -0.463 | 0.433 | 0.517 | .973** | 0.114 | .686* | -0.557 | .827** | -.630* | .693* |
| MeIQ | .824** | .869** | 1 | -.764** | 0.117 | .776** | .949** | -0.213 | 0.411 | -.818** | .730** | -.729** | .898** |
| Harman | -.817** | -0.463 | -.764** | 1 | 0.292 | -.722** | -.612* | .663* | 0.153 | .885** | -0.444 | .616* | -.710** |
| Norharman | -0.096 | 0.433 | 0.117 | 0.292 | 1 | 0.099 | 0.325 | .877** | .896** | 0.341 | 0.289 | 0.053 | 0.153 |
| MeAαC | .923** | 0.517 | .776** | -.722** | 0.099 | 1 | .678* | -0.112 | 0.325 | -.612* | 0.344 | -0.545 | .904** |
| Polar HAs | .710** | .973** | .949** | -.612* | 0.325 | .678* | 1 | -0.006 | .607* | -.673* | .792** | -.675* | .821** |
| Nonpolar HAs | -0.332 | 0.114 | -0.213 | .663* | .877** | -0.112 | -0.006 | 1 | .792** | .649* | -0.024 | 0.293 | -0.104 |
| Total HAs | 0.17 | .686* | 0.411 | 0.153 | .896** | 0.325 | .607* | .792** | 1 | 0.105 | 0.464 | -0.179 | 0.419 |
| Glucose | -.740** | -0.557 | -.818** | .885** | 0.341 | -.612* | -.673* | .649* | 0.105 | 1 | -.619** | -.456* | -.671* |
| Creatinine | 0.374 | .827** | .730** | -0.444 | 0.289 | 0.344 | .792** | -0.024 | 0.464 | -.619** | 1 | .544** | 0.509 |
| Creatine | -0.532 | -.630* | -.729** | .616* | 0.053 | -0.545 | -.675* | 0.293 | -0.179 | -.456* | .544** | 1 | -.602* |
| Free radical | .900** | .693* | .898** | -.710** | 0.153 | .904** | .821** | -0.104 | 0.419 | -.671* | 0.509 | -.602* | 1 |
